# Baseline bony erosions and time-averaged DAS28 predict discontinuation of TNF inhibitors in rheumatoid arthritis

**DOI:** 10.1038/s41598-022-24027-6

**Published:** 2022-11-19

**Authors:** Hong Ki Min, Se Hee Kim, Sang-Heon Lee, Hae-Rim Kim

**Affiliations:** 1 Division of Rheumatology, Department of Internal Medicine, Konkuk University Medical Center, 120-1 Neungdong-Ro, Gwangjin-Gu, Seoul, Republic of Korea; 2 Division of Rheumatology, Department of Internal Medicine, Research Institute of Medical Science, Konkuk University Medical Center, Konkuk University School of Medicine, 120-1 Neungdong-Ro, Gwangjin-Gu, Seoul, Republic of Korea

**Keywords:** Immunology, Medical research, Risk factors

## Abstract

The present study evaluated the predictive role of baseline radiographic change and disease activity on drug retention and clinical response in patients with rheumatoid arthritis (RA) treated with tumor necrosis factor inhibitor (TNFi). Korean Observational Study Network for Arthritis (KORONA) registry was evaluated to identify RA patients treated with a TNFi. Disease activity score-28 (DAS28) was evaluated at baseline and 1 year after TNFi initiation or at termination of TNFi due to inefficacy (within 1 year). The retention rate of TNFi was compared in patients with and without bony erosions. The hazard ratio (HR) for drug retention was evaluated by Cox regression analysis, as was the odds ratio (OR) for achieving remission (DAS28 < 2.6). This study included 109 RA patients, including 97 (89%) women and 30 (27.5%) with erosions, who were treated with a TNFi. Higher baseline DAS28 was negatively associated with achievement of remission (OR = 0.56, 95% CI 0.35–0.88). The TNFi retention rate was significantly lower in RA patients with than in those without erosions (*p* = 0.04). Factors significantly associated with drug discontinuation included the presence of erosions (HR = 2.45, 95% CI 1.08–5.51) and higher time-averaged DAS28 (HR = 2.17, 95% CI 1.47–3.20), whereas concomitant methotrexate was associated with lack of drug discontinuation (HR = 0.40, 95% CI 0.17–0.95). The presence of erosions and high time-averaged disease activity could predict poor retention of TNFi by RA patients. Higher baseline DAS28 was associated with a reduced clinical response in patients with RA.

**Trial registration** Clinical Research Information Service of South Korea https://cris.nih.go.kr: KCT0000086, registered May 26, 2009.

## Introduction

Rheumatoid arthritis (RA) is an autoimmune-mediated systemic arthritis, with proper management needed to improve symptoms and prevent structural damage to joints^1^. Initial therapy consists of treatment with conventional synthetic disease-modifying antirheumatic drugs (csDMARDs), singly or in combination, but about 40–50% of RA patients fail to respond to these csDMARDs^2,3^. Guidelines of the European League Against Rheumatism (EULAR), the American College of Rheumatology (ACR), and the Korean College of Rheumatology (KCR) recommend the use of biologic (bDMARDs) or targeted synthetic (tsDMARDs) drugs in patients who fail to respond to csDMARDs^4–6^. Tumor necrosis factor inhibitors (TNFis) are the most widely used class of bDMARDs, with five TNFis, etanercept, adalimumab, infliximab, golimumab, and certolizumab pegol, currently approved for the treatment of RA. TNFis have shown better clinical efficacy than csDMARDs and improved the prognosis of extra-articular manifestations of RA, such as cardiovascular disease^7,8^.

RA is characterized by erosion, joint space narrowing (JSN), subluxation, and juxta-articular osteopenia^1^. Reduction of disease activity is the primary endpoint of clinical trials in RA^9^. The EULAR has defined a good response as a > 1.2 point reduction of disease activity score-28 joints (DAS28), and remission as the achievement of a DAS28 < 2.6^10^. In addition to reducing disease activity, another important treatment target in RA is the prevention of joint destruction, as joint destruction is irreversible and significantly reduces patient quality of life^11^. Long-term TNFi treatment in patients with early RA was found to slow radiographic structural damage^12^, and the achievement of remission by TNFi treatment showed beneficial effects on the radiographic progression of RA^13^. To date, however, the effect of baseline radiographic destruction in RA on TNFi retention rate has not been determined.

Real-world data have both advantages and disadvantages when compared with randomized clinical trials (RCTs). The most important advantage of real-world data is the inclusion of a wider variety of patients, including those with intractable disease. The heterogeneity of these populations can be an obstacle when evaluating the net effect of a specific medication on disease progression, but can also increase overall knowledge when considered along with RCTs^14^. In addition, most RCTs have limited follow-up duration, whereas real-world studies are observational, with generally longer total follow-up duration^14^. The Korean Observational Study Network for Arthritis (KORONA) registry enrolled only RA patients in Korea who were treated at university-based tertiary hospitals^15,16^. Data collected annually by the KORONA registry have included vast amounts of RA-related information, such as DAS28, HAQ, laboratory data, and radiographic changes of the hands and feet.

The present study aims to find predictors of TNFi discontinuation, and achievement of remission (DAS28 < 2.6).

## Patients and methods

### Data source

**Figure 1:**
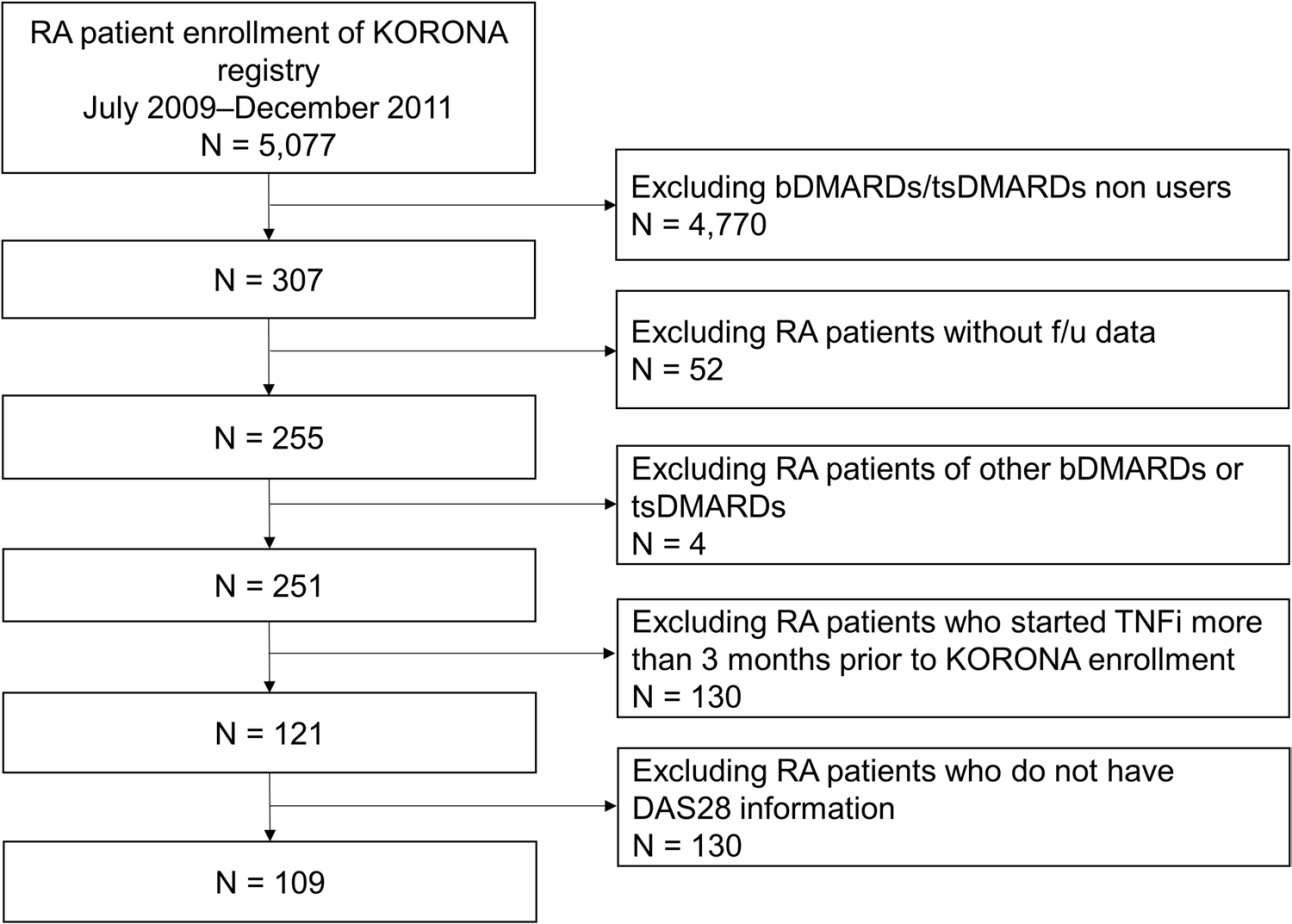
Flow chart for inclusion and exclusion of enrolled patients. *RA* rheumatoid arthritis, *KORONA* Korean Observational Study Network for Arthritis, *bDMARD* biologic disease-modifying antirheumatic disease, *tsDMARD* targeted synthetic disease-modifying antirheumatic drug, *TNFi* tumor necrosis factor inhibitor, *DAS28* disease activity score-28 joints.

The KORONA registry, established by the Clinical Research Center for Rheumatoid Arthritis (Hanyang University Hospital), enrolled RA patients from 23 university-based tertiary hospitals from July 2009 to December 2011 (Clinical Research Information Service of South Korea approval number: KCT0000086), and annual data collection was done until February 2017. The study was designed as prospective observational cohort study. Patients were included if they fulfilled the 1987 ACR classification criteria^17^, were aged > 18 years, and initiated or restarted TNFi treatment within 3 months of enrollment in the KORONA registry. Patients without disease activity parameters or follow-up data were excluded, as were patients treated with bDMARDs or tsDMARDs other than TNFis, and patients who initiated or restarted TNFi treatment more than 3 months prior to KORONA registry enrollment (Fig. 1). The reporting of this study conforms to the STROBE statement^18^.

### Data collection and outcomes

Demographic and clinical features, laboratory and clinical data, and health-related outcomes were collected at baseline and annually^15,16^. DAS28 was calculated based on the erythrocyte sedimentation rate (ESR), tender joint count, swollen joint count, and visual analog scale (VAS) for patient’s global assessment (PtGA)^19^. Smoking status at baseline was recorded as nonsmoker, ex-smoker, or current smoker. Regular exercise was recorded as a dichotomous variable (yes or no) by asking whether the patients exercise at least 3 times a week. The presence of erosions or JSN on the hands or feet was determined radiographically at baseline. Swollen and tender joints were assessed by physical examination. The VAS for physician’s global assessment (PhGA) and PtGA each ranged from 0 to 100. ESR (mm/hr), C-reactive protein (CRP) concentration (mg/dL), and positivity for rheumatoid factor (RF) and anticitrullinated peptide Ab (ACPA) were recorded at enrollment. The time-averaged DAS28 and health assessment questionnaire (HAQ) were calculated from their respective areas under the curve during total follow-up via dividing area under ROC curve of DAS28 and HAQ with total follow up days^16,20,21^. The area under ROC curve was calculated by using parameters (DAS28, HAQ) of each time points and duration between dates when each parameter was checked. Baseline medications were recorded, including nonsteroidal anti-inflammatory drugs (NSAIDs), daily doses of glucocorticoids, and csDMARDs (methotrexate, hydroxychloroquine, leflunomide, and sulfasalazine). Patients were followed-up until they discontinued TNFi or through the end of the KORONA registry study (until February 2017). The reason for TNFi discontinuation was also recorded.

### Statistical analyses

Continuous data were assessed using the Kolmogorov–Smirnov test to determine the normality of distribution, with normally distributed data reported as the mean ± SD and non-normally distributed data as the median with interquartile range. Continuous variables were compared with Student T test or Mann Whitney U test. Categorical variables were expressed as numbers and percentages and compared by chi-square test. The Kaplan–Meier method was utilized to determine the cumulative incidence of TNFi discontinuation due to inefficacy, with incidence in RA patients with and without baseline erosions compared using log-rank tests. Logistic regression analysis was performed to find predictors of achieving clinical remission. All variables with *p* value under 0.05 in univariate logistic regression analysis were included in multivariate logistic regression analysis, except for variable with variance inflation factor > 10. TNFi discontinuation due to other causes than inefficacy was censored. Factors associated with the inefficacy of TNFi were evaluated by univariate Cox proportional regression analyses and presented as hazard ratios (HRs) and 95% confidence intervals (CI). Variables with *p*-values < 0.05 on univariate analyses, except for multicollinear variables, were included in multivariate Cox regression analysis. All statistical analyses were performed using R software (R for Windows 3.3.2; The R Foundation for Statistical Computing, Vienna, Austria), with *p*-values < 0.05 considered statistically significant.

### Ethics approval and consent to participate

The present study conformed to the Declaration of Helsinki and Good Clinical Practice guidelines and was approved by the institutional review boards of each participating hospital. Written informed consent was obtained from each patient prior to enrollment. The KCR provided data from the KORONA registry, and approval for analysis was obtained from the Institutional Review Board of Konkuk University Medical Center (2020-05-053).

## Results

### Baseline characteristics of RA patients prior to TNFi initiation

Patients who restarted a TNFi after previous treatment with a TNFi, however, were included. Median patient age was 52.0 years; 97 (89%) patients were women, and 12 (11%) were men. At baseline, bony erosions was present in 30 (27.5%) patients and JSN in 39 (35.8%). Ninety-two (84.4%) patients concomitantly used methotrexate, and 13 (11.9%) had been previously treated with a TNFi. Ninety (82.6%) patients were positive for RF, and 79 (72.5%) were positive for ACPA. Other baseline characteristics are presented in Table 1.

**Table 1:** Baseline characteristics of enrolled RA patients.

	RA patients (N = 109)
Age (years)	52.0 [46.0; 63.0]
Female (n, %)	97 (89.0%)
Disease duration (years)	2.0 [1.0; 6.1]
BMI (kg/m^2^)	22.4 [20.5; 24.7]
Regular exercise (yes)	47 (43.1%)
**Smoking**	
Nonsmoker	97 (89.0%)
Ex-smoker	6 (5.5%)
Current smoker	6 (5.5%)
Baseline Td joint (yes)	17 (15.6%)
Baseline swollen joint (yes)	22 (20.2%)
Baseline erosions of hands or feet	30 (27.5%)
Baseline JSN of hands or feet	39 (35.8%)
**Medication**	
Methotrexate	92 (84.4%)
Hydroxychloroquine	5 (4.6%)
Sulfasalazine	1 (0.9%)
Leflunomide	2 (1.8%)
NSAIDs	92 (86.8%)
Glucocorticoid dose (mg/day, equivalent to prednisolone)	2.5 [2.0; 5.0]
Previous bDMARDs use history (yes)	13 (11.9%)
Adalimumab	6
Etanercept	4
Infliximab	3
**Current TNFi**	
Adalimumab	48 (44.0%)
Etanercept	61 (56.0%)
**Laboratory findings**	
CRP (mg/dL)	0.4 [0.3; 1.1]
ESR (mm/hr)	26.0 [13.0; 45.0]
RF positive	90 (82.6%)
ACPA positive	79 (72.5%)
PhGA (0–100)	22.0 [11.0; 36.0]
PtGA (0–100)	32.0 [13.0; 57.0]
Baseline DAS28	4.0 [3.2; 5.0]
Baseline HAQ	0.8 [0.4; 1.2]

All data are presented as number (%) or median with interquartile range.

*RA* rheumatoid arthritis, *BMI* body mass index, *JSN* joint space narrowing, *NSAID* nonsteroidal anti-inflammatory drug, *bDMARDs* biological disease-modifying antirheumatic drugs, *TNFi* tumor necrosis factor inhibitor, *CRP* C-reactive protein, *ESR* erythrocyte sedimentation rate, *RF* rheumatoid factor, *ACPA* anticitrullinated protein antibody, *PhGA* physician’s global assessment, *PtGA* patient’s global assessment, *DAS28* disease activity score 28, *HAQ* health assessment questionnaire.

### Achieving remission in TNFi users

Evaluation of DAS28 at baseline and after 1 year, or at the time of TNFi discontinuation in patients who stopped treatment, showed that 31 (28.4%) patients, 26 women and five men, achieved remission (DAS28 < 2.6). Univariate logistic regression analysis showed that baseline PtGA (odds ratio [OR] = 0.98, 95% confidence interval [CI] 0.96–0.99), DAS28 (OR = 0.49, 95% CI 0.31–0.71), and HAQ (OR = 0.31, 95% CI 0.13–0.66) were significantly associated with the achievement of remission. Multivariate analysis, however, showed that baseline DAS28 (OR = 0.56, 95% CI 0.35–0.88) was the only factor independently associated with the achievement of remission (Table 2).

**Table 2:** Univariate and multivariate logistic regression analysis of factors associated with the achievement of remission (DAS28 < 2.6) in patients with RA.

	Univariate		Multivariate^†^	
	OR	95% CI	OR	95% CI
Age	0.99	0.96–1.02		
Disease duration (year)	0.90	0.79–1.00		
BMI	1.09	0.96–1.24		
Baseline erosions of hands or feet	0.54	0.18–1.42		
Baseline JSN of hands or feet	0.42	0.15–1.05		
Baseline PtGA (0–100)	0.98*	0.96–0.99	0.99	0.97–1.02
Baseline PhGA (0–100)	0.98	0.96–1.00		
Baseline Td joint (yes)	0.68	0.23–2.62		
Baseline swollen joint (yes)	0.49	0.18–1.33		
Baseline DAS28	0.49***	0.31–0.71	0.56*	0.35–0.88
Baseline HAQ	0.31**	0.13–0.66	0.57	0.21–1.39
**Smoking**				
Nonsmoker	(reference)			
Ex-smoker	1.30	0.17–7.05		
Current smoker	0.77	0.13–5.46		
Regular exercise (yes)	1.35	0.58–3.13		
Rheumatoid factor positive	1.61	0.53–6.04		
ACPA positive	1.13	0.45–3.03		
Baseline ESR (mm/h)	0.95***	0.93–0.98		
Baseline CRP (mg/dL)	0.77*	0.12–0.74		
Previous bDMARDs use	0.42	0.06–1.69		
Methotrexate	2.04	0.61–9.36		
Glucocorticoid dose (mg/day, equivalent to prednisolone)	1.03	0.85–1.25		
NSAID	0.66	0.14–2.30		
Female	0.51	0.15–1.86		

*OR* odds ratio, *CI* confidence interval, *RA* rheumatoid arthritis, *BMI* body mass index, *JSN* joint space narrowing, *NSAID* nonsteroidal anti-inflammatory drug, *bDMARDs* biological disease-modifying antirheumatic drugs, *TNFi* tumor necrosis factor inhibitor, *CRP* C-reactive protein, *ESR* erythrocyte sedimentation rate, *RF* rheumatoid factor, *ACPA* anticitrullinated protein antibody, *PhGA* physician’s global assessment, *PtGA* patient’s global assessment, *DAS28* disease activity score 28, *HAQ* health assessment questionnaire.

^†^All variables with *p*-values < 0.05 in univariate logistic regression analyses were included in multivariate analysis, except for variables with variance inflation factor > 10.

*p < 0.05, **p < 0.01, ***p < 0.001.

### Reasons and predictors of TNFi discontinuation

**Figure 2:**
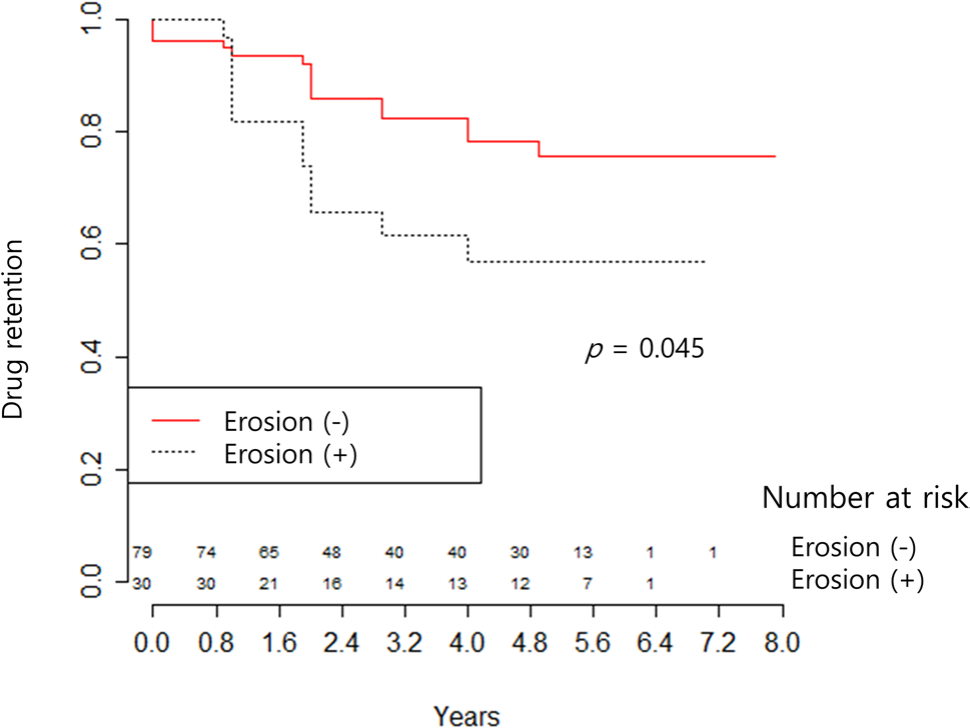
Kaplan–Meier analysis of TNFi retention rates in RA patients with and without baseline erosions of the hands or feet. Erosions (+), RA patients with baseline erosions of the hands or feet; Erosions (−), RA patients without baseline erosions of the hands or feet.

Forty-four TNFi discontinuations occurred during 370.1 person-years (PYs) of the study. Reasons for TNFi discontinuation included inefficacy (n = 26), side effects (n = 4), improvements in RA symptoms (n = 2), patient desire (n = 1), and other reasons (n = 11). Only TNFi discontinuation events due to inefficacy were included in drug retention analysis. Kaplan–Meier analysis showed that drug retention rate was significantly lower in RA patients with than without baseline erosions (Fig. 2, *p* = 0.04).

Cox regression analysis was performed to assess the associations of each variable with TNFi discontinuation due to inefficacy. Univariate analysis showed that baseline erosions (HR = 2.19, 95% CI 1.00–4.77), JSN (HR = 2.17, 95% CI 1.00–4.68), PtGA (HR = 1.03, 95% CI 1.01–1.04), PhGA (HR = 1.04, 95% CI 1.02–1.06), time-averaged DAS28 (HR = 2.93, 95% CI 2.09–4.11), and time-averaged HAQ (HR = 2.79, 95% CI 1.66–4.69) were significantly associated with increased risk of TNFi discontinuation, whereas treatment with MTX was significantly associated with a reduced risk of TNFi discontinuation (HR = 0.31, 95% CI 0.14–0.69). HR of sex was not calculated because none of the male RA patients discontinued TNFi due to inefficacy. The HR for TNFi discontinuation between monoclonal antibody form of TNFi and TNF receptor-Fc portion of IgG (etanercept) was not significant (data not shown). Multivariate Cox regression analysis included all variables with significance, except for those with multicollinearity (baseline erosions and JSN). Multivariate regression analysis was performed using two models, with model 1 including the presence of erosions and model 2 including the presence of JSN. In model 1, baseline erosions (HR = 2.45, 95% CI 1.08–5.51) and higher time-averaged DAS28 (HR = 2.17, 95% CI 1.47–3.20) were significantly associated with increased risk, and concomitant MTX (HR = 0.40, 95% CI 0.17–0.95) was significantly associated with a reduced risk, of TNFi discontinuation. In model 2, only time-averaged DAS28 was significantly associated with TNFi discontinuation, whereas JSN was not significant (Table 3).

**Table 3:** Univariate and multivariate Cox proportional regression analysis of factors associated with TNFi discontinuation in patients with RA.

	Univariate		Multivariate (model 1)^†^		Multivariate (model 2)^‡^	
	HR	95% CI	HR	95% CI	HR	95% CI
Age	0.99	0.97–1.03				
Disease duration (year)	0.99	0.90–1.08				
BMI	0.92	0.81–1.05				
Baseline erosions of hands or feet	2.19*	1.00–4.77	2.45*	1.08–5.51		
Baseline JSN of hands or feet	2.17*	1.00–4.68			2.22	0.97–5.10
Baseline PtGA (0–100)	1.03***	1.01–1.04	1.02	0.99–1.04	1.01	0.99–1.03
Baseline PhGA (0–100)	1.04***	1.02–1.06	1.01	0.99–1.04	1.02	0.99–1.04
Baseline Td joint (yes)	0.37	0.09–1.57				
Baseline swollen joint (yes)	0.27	0.06–1.14				
Time-averaged DAS28	2.93***	2.09–4.11	2.17***	1.47–3.20	2.28***	1.52–3.41
Time-averaged HAQ	2.79***	1.66–4.69	1.19	0.55–2.56	1.03	0.48–2.18
**Smoking**						
Nonsmoker	(reference)					
Ex-smoker	NA					
Current smoker	0.70	0.09–5.17				
Regular exercise (yes)	1.55	0.72–3.35				
Rheumatoid factor positive	1.00	0.38–2.66				
ACPA positive	1.37	0.55–3.40				
Baseline ESR (mm/h)	1.02***	1.01–1.04				
Baseline CRP (mg/dL)	1.04	0.88–1.23				
Previous bDMARDs use	0.87	0.26–2.89				
Etanercept (compared to adalimumab)	0.70	0.32–1.50				
Methotrexate	0.31**	0.14–0.69	0.40*	0.17–0.95	0.44	0.18–1.08
Glucocorticoid dose (mg/day, equivalent to prednisolone)	1.04	0.88–1.24				
NSAID	1.37	0.41–4.58				
Female sex	NA					

*HR* hazard ratio, *CI* confidence interval, *RA* rheumatoid arthritis, *BMI* body mass index, *JSN* joint space narrowing, *NSAID* nonsteroidal anti-inflammatory drug, *bDMARDs* biological disease-modifying antirheumatic drugs, *TNFi* tumor necrosis factor inhibitor, *CRP* C-reactive protein, *ESR* erythrocyte sedimentation rate, *RF* rheumatoid factor, *ACPA* anticitrullinated protein antibody, *PhGA* physician’s global assessment, *PtGA* patient’s global assessment, *DAS28* disease activity score 28, *HAQ* health assessment questionnaire.

*p < 0.05, **p < 0.01, ***p < 0.001.

^†^All variables with *p*-values < 0.05 in univariate Cox regression analyses, except for JSN, were included in multivariate analysis.

^‡^All variables with *p*-values < 0.05 in univariate Cox regression analyses, except for erosions, were included in multivariate analysis.

## Discussion

The present study showed that RA patients with bony erosions and higher time-averaged DAS28 have higher risk for TNFi discontinuation. These findings support the importance of controlling disease activity in aspect of drug retention, and possible poor retention rate of TNFi in RA patients with baseline joint destruction.

In present study, higher time-averaged DAS28 and baseline bony erosions were found as predictors for TNFi discontinuation for the first time. Several factors have been shown to affect the rate of bDMARD continuation. For example, bDMARD subtype^22,23^, sex^22–24^, baseline HAQ^22^, disease duration^25,26^, and concomitant csDMARDs^22^ have been associated with TNFi retention rate in RA patients. Although several studies found that etanercept had a higher retention rate than adalimumab or infliximab^22,23,26,27^, however, the present study did not show significant difference between monoclonal antibody form of TNFi and etanercept. Concomitant use of MTX was found to reduce the risk of TNFi discontinuation^24,26,27^. Female sex was found to significantly increase HR for TNFi discontinuation^22^, with one meta-analysis showing that the pooled HR for TNFi discontinuation was increased in women^23^. In contrast, other studies found that sex was not significant in predicting drug retention of TNFi in RA patients^24–26^. Longer disease duration prior to TNFi initiation was found to result in a significantly lower HR for TNFi discontinuation^25,26^, whereas other studies showed that disease duration was not associated with TNFi retention^23,24^. Higher baseline HAQ score, an indicator of poor health-related function, was reported to be associated with an increased risk of TNFi discontinuation^22^, whereas baseline DAS28 was not^24,26^. One study showed that DAS28 score at 6 months after TNFi initiation could predict long term drug response^28^. However, none of the studies included time-averaged DAS28 and HAQ in their analysis of TNFi retention rate in patients with RA. In the present study, disease duration and sex were not significantly associated with TNFi discontinuation, and time-averaged HAQ was significant only in univariate Cox regression analysis. A study from Spanish demonstrated that RA patients with multiple bDMARDs non-response were positively associated with baseline bony erosions^29^. Although previous studies analyzed factors associated with TNFi discontinuation in RA, those studies did not analyze the impact of radiographic joint destruction or time-averaged parameters on TNFi retention. In the present study, erosions in the hands or feet and higher time-averaged DAS28 score were associated with increased risk of TNFi discontinuation due to inefficacy. Moreover, consistent with earlier findings^24,26,27^, the present study found that the concomitant use of MTX lowered the risk of TNFi discontinuation in RA patients.

The present study found that higher baseline DAS28 score was associated with a significantly reduced likelihood of achieving remission, other factors, such as sex, age, BMI, disease duration, ESR/CRP, and HAQ, were not significantly associated with clinical response. An evaluation of clinical response of RA patients to etanercept treatment found that age < 49 years, body mass index (BMI) > 28.5 kg/m^2^, longer disease duration, and lower ESR/PhGA/Td joint count/HAQ were associated with achievement of remission at week 24^30^. Moreover, remission at week 128 was significantly associated with younger age, higher mental health scale score on the SF-36, higher swollen joint counts, and lower pain VAS^30^. Male gender has been associated with the likelihood of achieving good EULAR response^31^. Moreover, the percentage of patients achieving good EULAR response 12 months after TNFi initiation was lower in patients with high (baseline DAS28 > 5.1) than moderate (baseline DAS28 3.2–5.1) disease activity^31^. Nonresponse to TNFi has been observed more frequently in obese than in nonobese patients^32^, and the probability of remission was found to be lower in patients with higher than lower ESR^33^. Additional studies including a larger sample size and longer duration are needed to determine the baseline characteristics associated with clinical response to TNFis in RA patients.

The present study had several limitations. First, the results were drawn from observational real-world data (KORONA registry), which have intrinsic limitations when compared with RCTs. Second, about 90% of enrolled patients were female. Sex is an important factor in predicting drug retention and achieving clinical response in RA, but sex did not show significant difference on drug retention nor achieving clinical remission in the present study, primarily because the number of males in this study was small, as were the events being analyzed (i.e., TNFi discontinuation due to inefficacy or remission). Moreover, most of the enrolled RA patients were nonsmokers, limiting the ability to determine the effects of smoking status on drug retention and clinical response. Third, the KORONA registry was started in 2009, but most of the TNFis were later approved for use in Korea, making the number of TNFi initiators relatively small. Fourth, other bDMARDs or tsDMARDs were not included in analysis because, in 2009, these agents were approved for use only in RA patients who failed one or more TNFis, thus limiting the numbers of patients in the KORONA registry who received other bDMRADs or tsDMARDs. Fifth, radiographic evidence of damage to the hands or feet was not assessed semiquantitatively, as the KORONA registry did not record modified total sharp scores^34^, the most widely used method of semiquantitatively evaluating joint destruction in patients with RA. Finally, although the present study only included RA patients who initiated or restarted TNFi within 3 months prior to enrollment in the KORONA registry, 41 (37.6%) of the 109 patients had started taking a TNFi prior to enrollment in the KORONA registry. Because these patients may have achieved good EULAR responses before baseline data were collected in the KORONA registry, good EULAR response was not analyzed in the present study. However, the present study also has some strengths: the first study (1) which used time-averaged parameters as a predictor on TNFi retention, and (2) revealed association between baseline bony erosions and TNFi retention.

## Conclusions

In conclusion, higher baseline DAS28 reduced the likelihood of achieving remission in RA patients treated with TNFis. Erosions on the hands or feet at baseline was associated with a lower TNFi retention rate, increasing the likelihood of drug discontinuation due to inefficacy. Higher time-averaged DAS28 was also associated with a significantly increased risk of TNFi discontinuation due to inefficacy, whereas concomitant use of MTX reduced the risk of TNFi discontinuation. Radiographic evidence of joint destruction and higher DAS28 may have negative effects on drug retention and clinical response to TNFis, whereas treatment with MTX may reduce the risk of TNFi discontinuation due to inefficacy in RA patients.

## Data Availability

All data generated during this study are available from the corresponding author on reasonable request.

## Abbreviations

RA: Rheumatoid arthritis
DMARD: Disease-modifying antirheumatic drug
EULAR: The European League Against Rheumatism
ACR: American College of Rheumatology
KCR: Korean College of Rheumatology
TNFi: Tumor necrosis factor inhibitor
JSN: Joint space narrowing
DAS28: Disease activity score-28 joints
RCT: Randomized clinical trial
KORONA: Korean Observational Study Network for Arthritis
ESR: Erythrocyte sedimentation rate
VAS: Visual analog scale
PtGA: Patient’s global assessment
PhGA: Physician’s global assessment
CRP: C-reactive protein
RF: Rheumatoid factor
ACPA: Anticitrullinated peptide Ab
HAQ: Health assessment questionnaire
HRs: Hazard ratios
CI: Confidence interval
OR: Odds ratio
